# Investigation of Early Warning Indexes in a Three-Dimensional Chaotic System with Zero Eigenvalues

**DOI:** 10.3390/e22030341

**Published:** 2020-03-17

**Authors:** Lianyu Chen, Fahimeh Nazarimehr, Sajad Jafari, Esteban Tlelo-Cuautle, Iqtadar Hussain

**Affiliations:** 1 School of Electrical and Information Engineering, Jiangsu University of Technology, Changzhou 213001, China; chenly4254@sina.com; 2 Department of Biomedical Engineering, Amirkabir University of Technology, 424 Hafez Ave., Tehran 15875-4413, Iran; fahimenazarimehr@yahoo.com (F.N.); sajadjafari83@gmail.com (S.J.); 3 Health Technology Research Institute, Amirkabir University of Technology, No. 350, Hafez Ave., Valiasr Square, Tehran 159163-4311, Iran; 4 Department of Electronics, Instituto Nacional de Astrofísica, Óptica y Electrónica (INAOE), Tonantzintla, Puebla 72840, Mexico; 5 Department of Mathematics, Statistics and Physics, Qatar University, Doha 2713, Qatar; iqtadarqau@gmail.com

**Keywords:** chaotic system, shannon entropy, kolmogorov-sinai entropy, bifurcation point

## Abstract

A rare three-dimensional chaotic system with all eigenvalues equal to zero is proposed, and its dynamical properties are investigated. The chaotic system has one equilibrium point at the origin. Numerical analysis shows that the equilibrium point is unstable. Bifurcation analysis of the system shows various dynamics in a period-doubling route to chaos. We highlight that from the evaluation of the entropy, bifurcation points can be predicted by identifying early warning signals. In this manner, bifurcation points of the system are analyzed using Shannon and Kolmogorov-Sinai entropy. The results are compared with Lyapunov exponents.

## 1. Introduction

Investigating chaotic flows is an important topic in nonlinear dynamics [1,2]. Many researches have been worked on the design of special chaotic systems [3,4,5]. This work helps researchers to reveal the mystery of chaotic dynamics [6,7,8]. Chaotic flows with a plane of equilibria [9], without equilibria [10], with circular equilibria [11], with a line of equilibria [12], and with a stable equilibrium [13], are some examples. Understanding fractional-order chaotic systems is more challenging [14,15]. The control of integer and fractional-order chaotic systems has been a hot topic [16,17]. Various dynamics of chaotic flows can be studied using the bifurcation diagram versus a bifurcation parameter [18,19]. Also, Lyapunov exponents have shown the various dynamics of a chaotic system [20,21]. The modeling of chaotic biological and physical real-world systems is an active area of research [22,23,24], and their dynamics can also be evaluated by computing Lyapunov exponents. 

Critical slowing down is a unique property that can predict bifurcation points [25]. For instance, by approaching a tipping point, a system’s dynamic becomes slower [26,27]. There are two types of features that can predict bifurcation points: Metric-based and model-based indicators [28]. Metric-based indicators use various features of time series to predict tipping points [25], while model-based indicators estimate some models from the time series and then predict tipping points using the model, as shown in [29]. Recently, the Lyapunov exponent was proposed as an indicator of tipping points [30]. However, for many years, tipping point indicators have only been able to predict bifurcation points of type period-1 [31]. Nowadays, some measures exist to predict other types of bifurcations, as shown in [25]. In this manner, the prediction of bifurcation points has been a hot topic, since they can cause a massive change to an unwanted or desired state in dynamical systems.

In this paper, we use various entropies to predict the bifurcation points of a new chaotic system. The rest of this manuscript is organized as follows: A new three-dimensional chaotic system is proposed in Section 2, and its dynamical properties are investigated. In Section 3, entropies of the chaotic system for changing the bifurcation parameter are studied to predict bifurcation points. The entropies are compared with the Lyapunov exponents of the given chaotic system. The paper is concluded in Section 4.

## 2. The Proposed Chaotic System and Its Properties

A three-dimensional chaotic flow that includes a single parameter is introduced herein as follows:

(1) $$\begin{array}{c} \begin{array}{c} \dot{x}=y\\ \dot{y}=z \end{array}\\ \dot{z}=0.1x^{2}-0.48y^{2}+axz \end{array}$$

**Figure 1:**
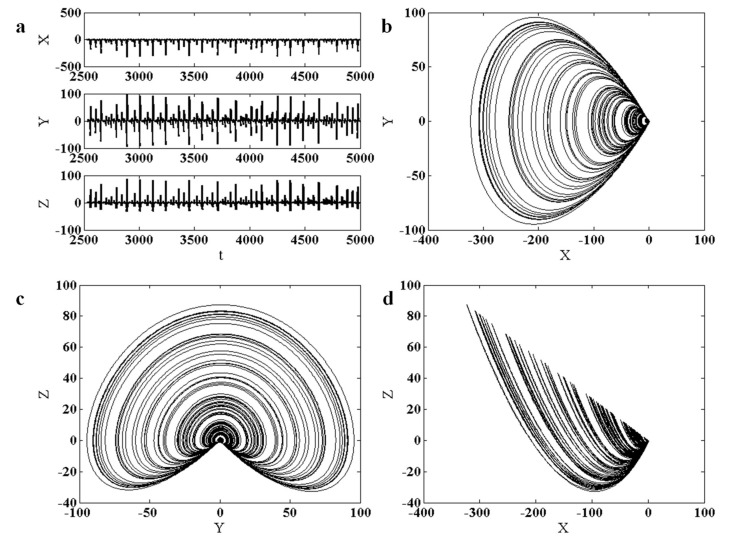
Time-series and phase-space projections of the chaotic attractor System (1), using a=0.37 and initial conditions $\left(x_{0},y_{0},z_{0}\right)=\left(-10.9,-12.04,33.68\right)$. (**a**) time-series; (**b**) projection in X−Y plane; (**c**) projection in Y−Z plane; and (**d**) projection in the X−Z plane. It can be seen that the attractor is symmetric around the line y=0.

The proposed chaotic system generates a chaotic attractor for a determined value of the bifurcation parameter. In this case, the bifurcation parameter in (1) is selected as a=0.37, and the attractor is generated using the initial conditions $\left(x_{0},y_{0},z_{0}\right)=\left(-10.9,-12.04,33.68\right)$. Lyapunov exponents of the chaotic attractor are evaluated as (0.017,0,−4.9408). The time-series of the three state variables and two-dimensional projections in the phase-spaces of the proposed chaotic attractor given in system (1) are presented in Figure 1. It can be seen that the chaotic attractor is symmetric around the line y=0. 

To calculate the equilibrium points of the chaotic system from (1), the derivatives of state variables must be set to zero. In this way, one obtains

(2) $$\begin{array}{c} \begin{array}{c} y=0\\ z=0 \end{array}\\ 0.1x^{2}-0.48y^{2}+axz=0 \end{array}$$

It is clear that the proposed chaotic system has one equilibrium point located at the origin. In this manner, to calculate the stability of the origin, the Jacobian matrix when a=0.37 is formulated as follows:

(3) $$Jac=\left[\begin{array}{ccc} 0 & 1 & 0\\ 0 & 0\; & 1\\ 0.2x+0.37z & -0.96y & 0.37x \end{array}\right]$$

From (3), the characteristic equation of the chaotic system in its equilibrium point at the origin becomes $\lambda^{3}=0$. As a result, the equilibrium at the origin has three zero eigenvalues. Zero eigenvalues cannot determine the stability of the equilibrium points. By performing numerical calculations, one finds that the origin is unstable. 

**Figure 2:**
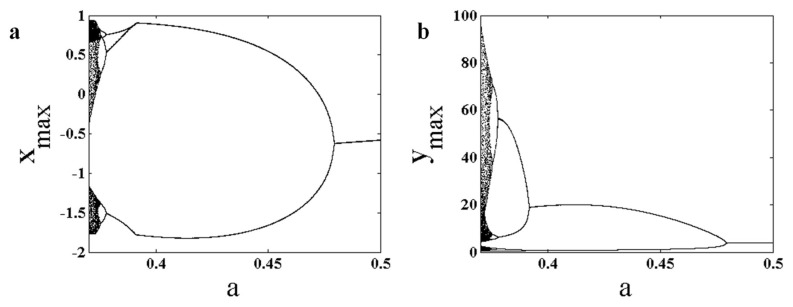

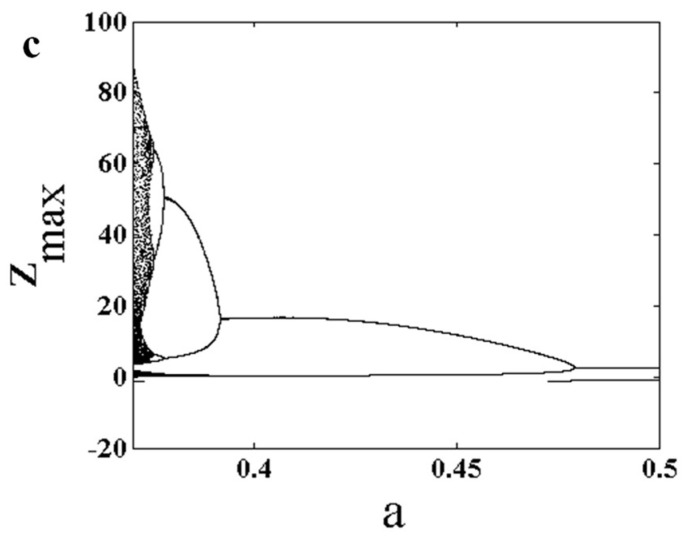
Bifurcation diagram of System (1) with respect to changing parameter a and forward continuation method; (**a**) bifurcation diagram of peak values of x variable; (**b**) bifurcation diagram of peak values of y variable; (**c**) bifurcation diagram of peak values of z variable.

Various dynamics of the chaotic system given in (1) are studied herein using the bifurcation diagram of each state variable with respect to the bifurcation parameter, as shown in Figure 2. The aim of this paper is to investigate predictions of bifurcation points in chaotic flows. So, one parameter is chosen with various bifurcations in a period-doubling route to chaos. The bifurcation diagrams have plotted peak values of x,y, and z variables by changing the control parameter in the range a∈[0.37, 0.5], and the forward continuation method was applied. The first initial conditions at a=0.37 are $\left(x_{0},\;y_{0},\;z_{0}\right)=\left(-10.9,-12.04,\;33.68\right)$, and in the following parameters, the initial conditions are selected from the end values of the attractor generated by using the previous parameter values. The chaotic system shows a period-doubling route to chaos by changing parameter a. From the viewpoint of maximum values of x variable, the system shows period-1 dynamic in the interval a∈[0.4795, 0.5], period-2 dynamic in a∈[0.3914, 0.4795], period-4 dynamic in the interval a∈[0.3778, 0.4795], and follows by a period-doubling route to chaos with decreasing parameter a.

## 3. Entropy Analysis as an Early Warning Signal

The unpredictability of time-series can be predicted using entropy measures, as shown in [32]. Boltzmann and Shannon were pioneers in defining entropy [33]. The famous definition of entropy can be evaluated using the mathematical expression given in (4), where $\rho_{i}$ is the probability of the i−th state.

(4) $$H=-\underset{i}{\sum }\rho_{i}\log\left(\rho_{i}\right)$$

In chaotic dynamical systems, another kind of entropy is applicable, known as Kolmogorov-Sinai entropy [34]. Its definition is based on the first Poincaré recurrence (FPRs) time, which is shown by $\tau_{i}$. This entropy is like that given in (5), where β is a D-dimensional box in the phase-space with side $\varepsilon_{1}$ where the FPRs are observed, and ρ(τ,β) is the probability distribution of $\tau_{i}$.

(5) $$H_{ks}\left(\beta \left[\varepsilon \right]\right)=\frac{1}{\tau_{min}\left(\beta \left[\varepsilon \right]\right)\;}\underset{\tau }{\sum }\rho \left(\tau ,\beta \left[\varepsilon \right]\right)\log\left(\frac{1}{\rho \left(\tau ,\beta \left[\varepsilon \right]\right)}\right)\;$$

To analyze the dynamics of the proposed chaotic system given in (1) and its bifurcation points, we extracted the Poincaré section of the attractors using its peak values. We then analyzed the complexity of the peak values. It should be considered that the slowness of the system close to the bifurcation points can be seen in the transient time, so we do not remove any transient time. This point should be noted in the simulations of this paper. In the study of slowing down, the transient time should not be removed, since the initial values are considered as a small disturbance. We should note that initial values are selected within the attractor’s range. 

**Figure 3:**
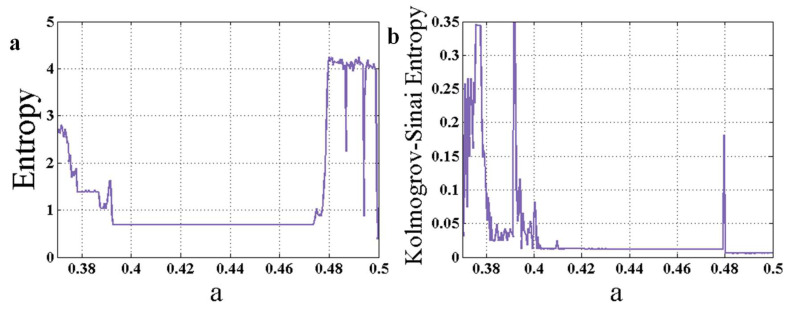
The entropy of the chaotic system (1), which is calculated using the peak values of the state variable *y*. (**a**) Shannon entropy for changing parameter a; (**b**) Kolmogorov-Sinai entropy for changing parameter a.

Part (a) of Figure 3 shows the entropy of the chaotic system (1), which is calculated using the peak values of the state variable y. In all simulations, the run-time for the attractors is 2000. The run-time is selected carefully in order to allow the system to achieve its stable state. Part (b) of Figure 3 shows Kolmogorov-Sinai entropy of the System with respect to changing parameter a. To investigate the quality of the indicators, we called four bifurcation points $BP_{1}:a=0.4795;\;BP_{2}:a=0.3919;\;BP_{3}:a=0.3779;\;BP_{4}:a=0.3755$. $BP_{1}$ is a bifurcation from period-2 to period-1 dynamics, $BP_{2}$ is a bifurcation from period-4 to period-2, $BP_{3}$ is from period-8 to period-4, and $BP_{4}$ is from period-16 to period-8. One can see in Figure 3 that all bifurcation points can be predicted using the two entropy measures. However, in part (a), in the interval a∈[0.48, 0.5], while the system has period-1 dynamic (as can be seen in part (b) of Figure 2), the entropy is high. This happens because the transient time of the period-1 dynamics is very high. A preliminary conclusion can be associated with the statement that Kolmogorov-Sinai entropy has many oscillations which can result in false-positive predictions. However, initial values are selected within the attractor’s range. It seems that the transient time of the period-1 dynamic is much higher than those of other dynamics. It should be considered that the forward continuation method is used in the calculations. So, the system is very close to stability, and the initial conditions represent a small disturbance. False positives exist because, with small disturbances, some attractors have much more transient time than the others; however, the simulations show that the small disturbance does not cause the solutions to go into unstable manifolds.

**Figure 4:**
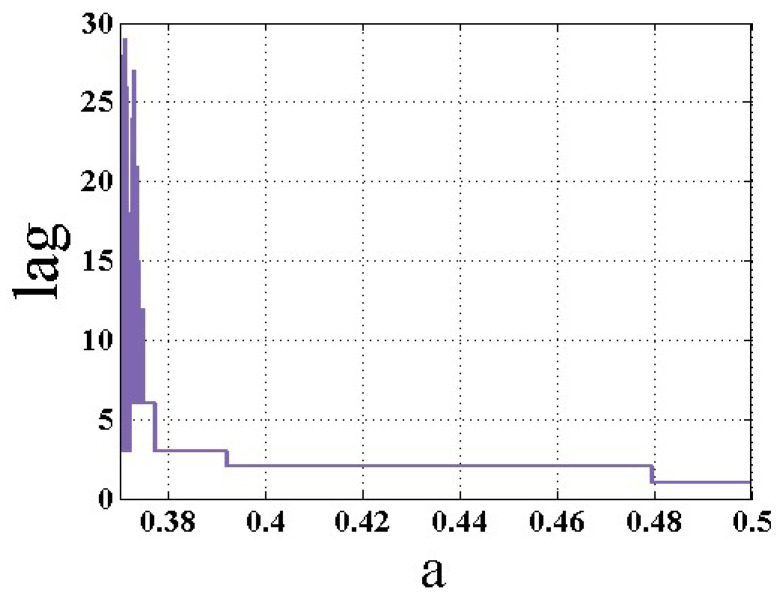
The extracted period of the chaotic system (1) for changing parameter (a).

**Figure 5:**
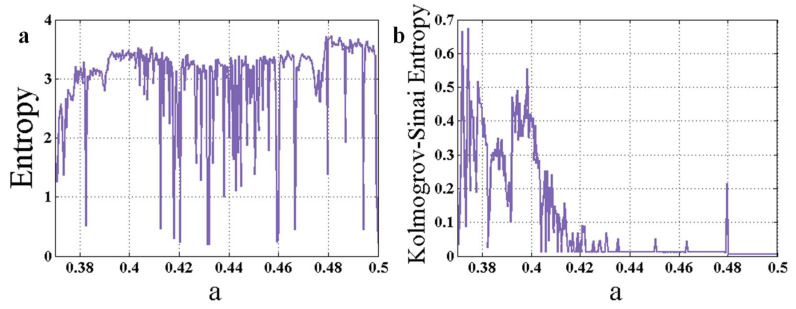
The mean value of the entropies in all vectors of cycles by changing parameter a; (**a**) Shannon entropy; (**b**) Kolmogorov-Sinai entropy.

In order to match the indicators with the prediction of various dynamics other than period-1, we use the method described in [25], but applied to the peak values of the proposed chaotic flow. Therefore, in the first step, the period of peak values signal is extracted using the autocorrelation function. In this algorithm, autocorrelation at various lags is calculated, and the minimum lag, which maximizes the autocorrelation, is selected as the period of the signal (m). In this paper, the maximum lag of autocorrelation is taken as 29. The transient parts are not helpful in detecting the final state, and can cause errors in extracting the type of dynamic. Therefore, these parts of the signal are removed by observing a long time-series. In other words, we used the maximum lag of autocorrelation as 29. This is a threshold whereby if the system is not periodic, it is obtained in the algorithm as the first nonzero maximum of the autocorrelation function among the different lags. The extracted period of the chaotic system (1) for changing parameter a is shown in Figure 4. In the second step, the m vectors are extracted in each cycle of the period m-cycle signal. The mean value of the entropies in all vectors are averaged, and the results are shown in Figure 5. The results show that the two modified entropies can predict the four bifurcation points $BP_{1}-BP_{4}$, but these measures are noisy, and there are many false positives. So, the entropies of the original methods are more valid than the new ones. Simulations with higher run-times show that the results are not dependent on the run-time, and that the selected run-time is enough.

**Figure 6:**
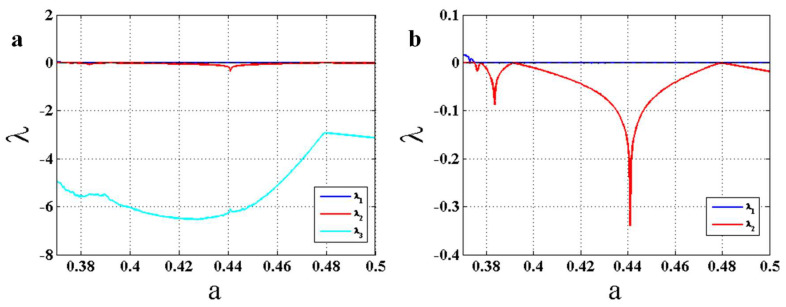
Lyapunov exponents of the chaotic system (1) using the forward continuation method; (**a**) three Lyapunov exponents; (**b**) the two largest Lyapunov exponents.

The Lyapunov exponents of the proposed chaotic system (1) using the forward continuation method are shown in Figure 6. Three Lyapunov exponents are shown in part (a), and for a better appreciation, the two largest Lyapunov exponents are presented in part (b) of the figure. The results show that in all bifurcation points, one Lyapunov exponent approaches zero, which makes it the most reliable indicator of bifurcation points.

## 4. Conclusions

A novel three-dimensional chaotic flow has been proposed in this paper. It has one equilibrium point at the origin with three zero eigenvalues. Such systems are very rare in the literature. Numerical simulations showed that the equilibrium is unstable. The chaotic attractor of the chaotic system and its various dynamics were discussed. Five methods of predicting tipping points were studied. Four methods were based on entropy, and the last one was a Lyapunov exponent. The first two methods were Shannon entropy and Kolmogorov-Sinai entropy. The results showed that Shannon entropy yields a false result in the period-1 dynamic, while the Kolmogorov-Sinai entropy does not. Then, the entropies were calculated on the extracted vectors of periods based on the type of dynamics. This method aimed to match the entropies with various dynamics in a period-doubling route to chaos. However, the results show that this method is not efficient in continuous systems, since it adds many false positives to the results. The last indicator was the Lyapunov exponents. That indicator did not have any false positives and approached zero at various bifurcation points. However, note that the calculation of Lyapunov exponents needs a long signal, while entropies do not.
